# Influence of Minor Cr Additions on Crystal Growth in Rapidly Solidified Al-20Zn Alloys

**DOI:** 10.3390/ma13020379

**Published:** 2020-01-14

**Authors:** Félix Royer, Julien Zollinger, Bernard Rouat, Michel Rappaz

**Affiliations:** 1 Department of Metallurgy & Materials Science and Engineering, Institut Jean Lamour, Université de Lorraine, Campus ARTEM, Allée André Guinier, F-54011 Nancy, France; felix.royer@univ-lorraine.fr (F.R.); bernard.rouat@univ-lorraine.fr (B.R.); 2 Laboratory of Excellence on Design of Alloy Metals for low-mAss Structures (DAMAS)—Université de Lorraine, F-57073 Metz, France; 3 Ecole Polytechnique Fédérale de Lausanne, Institute of Materials, Station 12, CH-1015 Lausanne, Switzerland; michel.rappaz@epfl.ch

**Keywords:** aluminium alloys, nucleation, ISRO, quasicrystals, solidification

## Abstract

It has been discovered quite recently that Icosahedral Short-Range Order (ISRO) of atoms in the liquid phase of metallic alloys surrounding some trace elements added to the melt can influence both the nucleation and growth of the primary phase. In this work, Al-20wt.%Zn alloys without and with 0.1 wt.% Cr additions have been processed using a free-falling droplet technique. This technique allows to undercool the liquid droplet during its fall and thus to have rapid directional solidification conditions when it collides a copper-cooled substrate. Under such rapid solidification conditions, microstructural and EBSD analyses have shown that, under such rapid solidification conditions, Cr addition is responsible for the nucleation and growth of feathery grains (or twinned dendrites). This morphology specific to aluminum alloys has been discovered more than seventy years ago without a clear identification of its origin. The angular analysis between twinned dendrites indicates a behavior similar to those of the propagation of topological defects, through an ISRO-induced stacking fault mechanism.

## 1. Introduction

Alloys with cubic crystal structure usually solidify with their dendrite trunks and arms growing along ⟨100⟩ directions. Aluminium alloys are known for exhibiting additional unusual dendrite growth morphologies, depending in particular on additional solute elements such as Zn [1] or Ge [2]. One dendritic growth morphology specific to aluminium alloys is the formation of twinned dendrites, within what are referenced as “feathery grains” [3,4,5,6]. The growth direction and propagation of twinned dendrites are fairly well understood: their trunk grows along a ⟨110⟩ direction and is split in its centre by a coherent {111} twin plane. However, the origin of such twinned structure remained unknown until the PhD work of Kurtuldu [7] on the effect of trace elements on the solidification of Al alloys brought a possible explanation.

Kurtuldu et al. [8] have studied the peculiar microstructure of Al-Zn alloys with and without Cr additions of 200 to 1000 ppm by weight (0.02–0.1 wt.%). Under equiaxed solidification conditions, these authors have noticed that chromium refines the grain structure and induces an abnormal density of twinned grain boundaries. Furthermore, Multiple Twin (MT) relationships between nearest-neighbours could only be explained if one considers the geometry of an icosahedron (or interlocked icosahedron) template on which face-centered cubic (fcc) grains form with the following heteroepitaxy relationships: 3-fold symmetry axes of icosahedron // ⟨111⟩ directions of fcc, and 2-fold symmetry axes of icosahedron ⊥ ⟨110⟩ directions of fcc.

The Al-Cr diagram shows the existence of the Al_7_Cr (also named Al_45_Cr_7_) intermetallic phase, which is a well-known approximant phase of AlCr quasicrystals (QC), with many icosahedral motifs in its large monoclinic unit cell. This phase remains unobserved in Al-Zn alloys below 2wt.%Cr, and it has been shown that the alloy undergoes a peritectic reaction, dissolving the Al_7_Cr phase during the peritectic reaction and transformation [9].

Icosahedral Short-Range Order (ISRO) of atoms in the liquid, already suggested by Frank in 1952 [10], happens as the local energy required to form this arrangement is lower than that of a plain random distribution of atoms because of a lower entropy [11]. It occurs thanks to localized covalent bonding between atoms. However, the central 5-fold symmetry of an icosahedron does not allow the creation of a crystal with translational symmetry, as the 5th order symmetry would ultimately lead to angular gaps on a large scale. This was the reason invoked by Frank for the interpretation of the large undercooling measured by Turnbull [12]. It has been shown later [13] that, as the undercooling is increased, the density of ISRO arrangements in the liquid also increases. It has also been suggested [14] that the addition of elements having a smaller covalent radius than the solvent can stabilize ISRO in the liquid. As a matter of fact, Cr in Al-Zn alloys satisfies this condition (which is necessary but definitely not sufficient) to form ISRO.

The so-called “QC-mediated” or “ISRO-mediated” nucleation of the fcc phase in Al alloys proposed by Kurtuldu et al. [8] is as follows: (1) ISRO in the liquid leads to (2) icosahedral QC (iQC) formation as the undercooling increases. The formation of iQC is favored as its interfacial energy with the liquid is at least one order of magnitude smaller than that of the fcc phase [15]. As the liquid becomes depleted in Cr, the fcc phase forms on the iQC’s with the abovementioned heteroepitaxy relationship. The twenty grains forming in this way on the facets of an icosahedron exhibit twinned or near-twin mutual relationships (The angle between the internal planes of the twenty tetrahedra of an icosahedron make an angle of 72 deg., while {111} tetrahedra of the fcc phase make an angle of 70.53 deg. This 1.47 deg. difference leads to near-twin relationships when they cumulate, as shown by Kurtuldu et al. [7].). Similar observations were made in yellow gold Au-12.5wt.%Cu-12.5wt.%Ag, with very small additions of iridium (10 to 200 ppm) [16].

In two recent papers [3,17], ISRO has been shown to also influence the growth directions of dendrites during solidification. In Al-20wt.%Zn alloys [3], ⟨100⟩ dendrites are replaced by ⟨110⟩ trunks at intermediate growth rate (<200 μm/s) when 1000 ppm Cr is added. At higher growth speed (1–2 mm/s), ⟨100⟩ trunks are retrieved but with the concurrent formation of ⟨110⟩ twinned dendrites. This effect was tentatively explained by an “ISRO-induced stacking fault” mechanism, i.e., ISRO motifs attaching to the growing fcc phase with or without annihilation of the induced stacking fault depending on the velocity. In pink gold Au-20.5wt.%Cu-4.5wt.%Ag, a similar effect was observed in the columnar zone of a small specimen which fell onto a copper-chill substrate (free-falling droplet (FFD) technique) [17]. Under such rapid solidification conditions, ⟨100⟩ dendrites are replaced by a ⟨111⟩ cellular-type microstructure growing along the thermal gradient direction. Moreover, a copper-rich phase was observed and seems to nucleate before the gold phase, as if resulting from a spinodal decomposition of the liquid, which is not the case without iridium.

Since rapid solidification exacerbates ISRO in gold alloys with Ir additions, an interesting point is to study how Al-Zn alloys would behave during the rapid solidification conditions of the FFD technique. This paper presents these results for Al-20wt.%Zn alloys without and with 0.1wt.%Cr. At the light of these results, a new mechanism for the nucleation and growth of twinned dendrites is proposed.

## 2. Materials and Methods

The two alloys were elaborated and cast by Constellium R&D Center in Voreppe, France, from high purity (99.999%) pure metals. The selected alloys composition is Al-20wt%Zn and Al-20wt.%Zn-0.1wt.%Cr (all the compositions will be given in weight percentages unless stated otherwise). Differential thermal analysis (DTA) was performed on both alloys. The solidus and liquidus for the Al-Zn and Al-Zn:Cr alloys are 585 °C and 586 °C, and 643 °C and 641 °C, respectively, which matches closely the Al-Zn phase diagram for a 20 wt.% zinc composition. It shows that the two ingots received are homogeneous and comply with the targeted composition, and that the minute addition of chromium does not modify significantly the phase equilibria.

The main experiment used in the present study is the Free-Falling Droplet (FFD). The apparatus is quite simple: an inductor is placed within a sealed enclosure, and the sample is put inside a silica tube of approximately 2 cm of internal diameter at the top, and 3 mm aperture at the bottom, as the tube in itself is placed inside the inductor. Beneath the inductor and silica tube is a water-cooled copper chill substrate with a small silica container to avoid the droplet to produce a splash effect when landing on the substrate. A vacuum pump is connected to the enclosure, which can be filled also with argon or helium-10% hydrogen. The temperature of the sample is measured with a two-colour wavelength pyrometer. To operate the machine, a primary vacuum is established. Then, the enclosure is filled with argon. The sample can be heated and melted inside of the silica tube. Once the desired temperature is reached, a blast of inert and deoxidizing gas He-5%H_2_ is sent towards the sample via the top of the tube, hence pushing the droplet to fall on the chill substrate. After landing on the substrate, very fast cooling conditions are achieved: the cooling rate is estimated to reach −10^4^ K/s near the contact surface and −10^2^ K/s near the top of the fallen droplet. For each alloy, three samples were prepared to ensure reproducibility.

After solidification, the samples were polished on Struers TegraPol-11 rotary polishers, at 300 rpm, with increasingly fine SiC abrasive papers (500, 800, 1200, 4000). Then, the samples were polished using a diamond suspension of 1 µm and 0.5 µm. Finally, the samples were finished with O-PS solution from Struers ©, an alumina suspension solution of 0.04 µm grains. The samples were always cleaned in an ultrasonic bath containing pure ethanol. Two electrolytic polishings were then made. The solution used was the A2 of Struers. A typical etching cycle was 4 V for 10 s and 25 V for 4 s. The microstructure was observed with an optical Axioplan 2 microscope equipped with a Zeiss Axiocam MRc5. The grain orientation analysis was made with an FEI Quanta 600 F FEG-SEM, equipped with a Nordlys high-speed EBSD camera. The data were collected and analysed with the Channel 5 software suite Oxford Instruments, High Wycombe, UK.

## 3. Results

**Figure 1:**
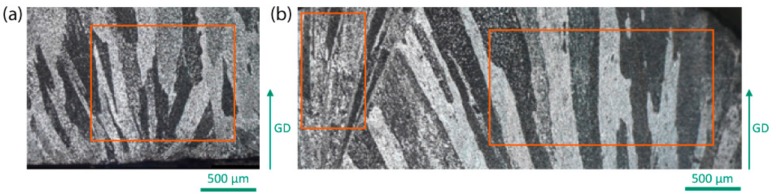
Microstructure of FFD samples: (**a**) for Al-20w.t%Zn alloy closed to the copper chill and (**b**) for Al-20wt.%Zn:0.1wt.%Cr at 1.5 mm from the copper chill. The rectangles indicate the position where EBSD analysis was done.

The droplets solidified with the FFD experiment were cut parallel to the temperature gradient, so as to observe the evolution of the microstructure from the contact with the copper substrate up to the top free surface. Figure 1 shows the microstructures of the Al-Zn (a) and Al-Zn:Cr (b) samples. Although Figure 1 only shows (a) the bottom region of the droplet in contact with the copper chill and (b) the region 1.5 mm from the copper chill, both samples exhibit a columnar zone over the whole thickness (3 mm). In the sample containing Cr (Figure 1b), a large feathery grain can be observed on the left side of the sample.

**Figure 2:**
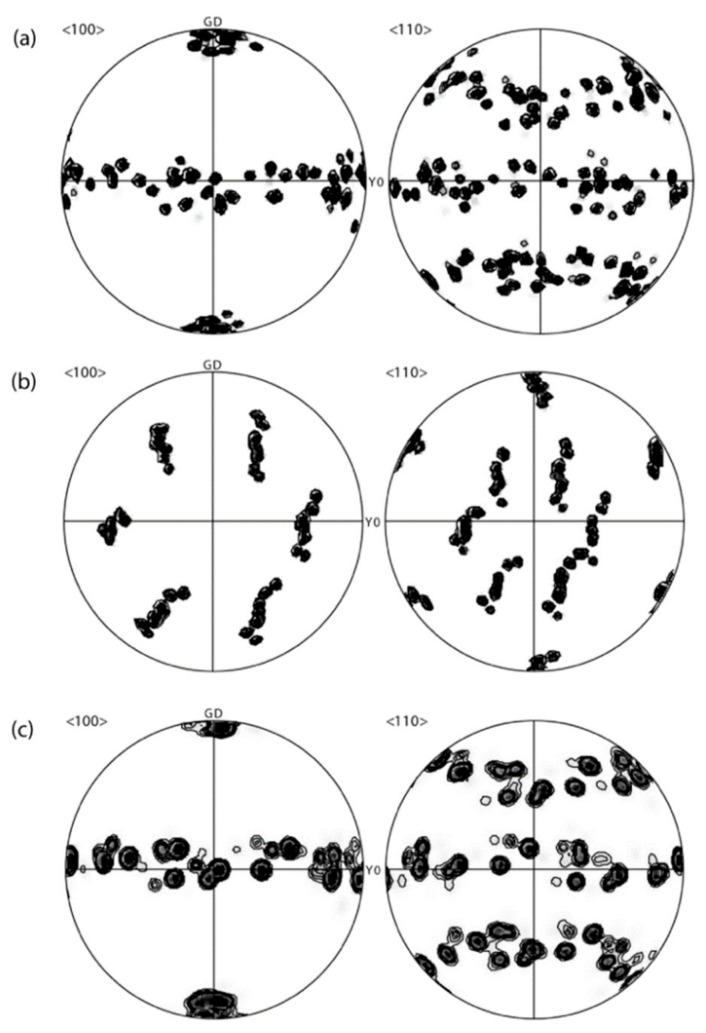
〈100〉 and 〈110〉 pole figures corresponding to rectangles shown in Figure 1 with the same orientation: (**a**) Al-Zn sample; (**b**) feathery grain; and (**c**) “regular” columnar zone in the Al-Zn:Cr sample. The thermal gradient (or growth) direction (GD) is vertical, as indicated on the pole figures.

The EBSD analysis performed on these samples is given in Figure 2a–c where the ⟨100⟩ and ⟨110⟩ pole figures are given, with the axes corresponding to those of Figure 1 (the thermal gradient is vertical in both figures). Figure 2a corresponds to the Al-Zn sample, and Figure 2b,c to the feathery grain and columnar zone of the Al-Zn:Cr sample, respectively. Due to the large grain size, only a limited number of grains (20 grains for columnar microstructures) are displayed in each pole figure, but the results are, however, consistent with the expected growth direction with respect to the thermal gradient. For both “regular” columnar zones (a) and (c), a dominant ⟨100⟩ texture is observed as a result of competition of ⟨100⟩ dendrites growing from nuclei formed at the bottom surface. The feathery grain seen on the left of Figure 2b exhibits the characteristic feature of a strong ⟨110⟩ texture parallel to the thermal gradient, confirming previous observations of ⟨110⟩ growth directions of twinned dendrites [4,5,6].

**Figure 3:**
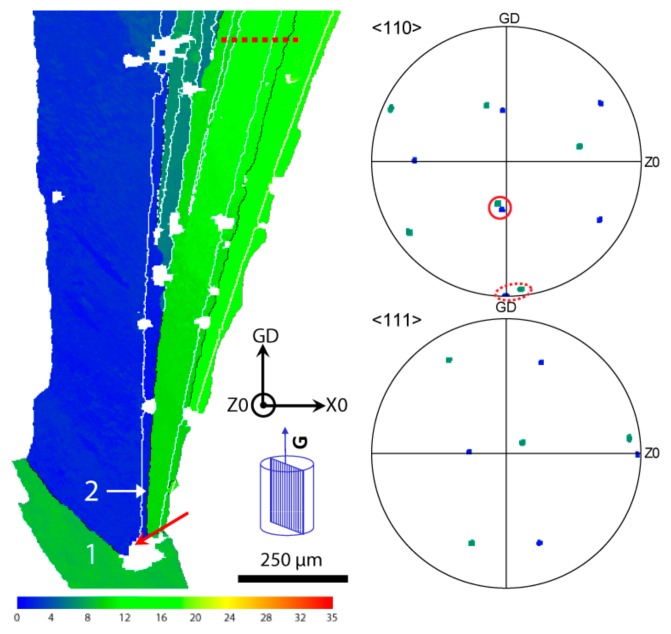
EBSD map of the feathery grain in a region close to its nucleation point. The colour corresponds to the misorientation between the thermal gradient direction (GD) and the closest ⟨110⟩ direction (see scale at the bottom). White lines correspond to twin or near-twin relationships. ⟨110⟩ and ⟨111⟩ pole figures of regions 1 and 2 are shown on the right with the two ⟨110⟩ growth directions circled with a dashed red ellipse, and their truly common ⟨110⟩ direction circled in red (see text).

A detailed EBSD analysis has been performed near the visible nucleation centre of the feathery grain. Figure 3 shows the false colour EBSD map reconstructed with the misorientation angle between the vertical thermal gradient (GD) and the common ⟨110⟩ growth direction of the twinned dendrites. As this is the common ⟨110⟩ direction of both sides of a twinned dendrite, they appear with the same colour in this representation. Except for a few white regions which could not be indexed by EBSD, one can see a green region labeled (1) and a blue one labeled (2). White lines indicate boundaries in twinning (or new-twinning) orientation relationship and the corresponding ⟨110⟩ and ⟨111⟩ pole figures are shown on the right for both regions with the same colour code.

In the bottom part of the figure is the parent regular dendritic grain (1) from which the feathery grain has “nucleated” (the nucleation center has been indicated with a red arrow). This grain has a ⟨110⟩ direction not precisely aligned with GD as can be seen in the dashed red ellipse of the corresponding pole figure. The first twins appear at the red arrow, in particular with a region 2 which is perfectly aligned with GD (blue corresponds to a 0 deg. misorientation with GD, while green extends from about 10 to 20 deg. misorientation with GD). Other twins continue to appear later during growth, mainly within the slightly misoriented green region. What is quite remarkable is that the blue region is in a near-twin relationship with the parent green grain on which it “nucleates”. Indeed, these two regions share a common ⟨110⟩ direction which is at 60 deg. from the not-perfectly aligned ⟨110⟩ growth direction. This common ⟨110⟩ direction is circled in red in the pole figure. In other words, the relative orientation of the blue and green regions is a combination of a perfect {111} twin plus a rotation around one of the three ⟨110⟩ common directions (red circle) which is not the growth direction (dashed red ellipse). This rotation around the common ⟨110⟩ is found to be 7.3 deg., i.e., the cumulated misfit of 5 tetrahedra of an icosahedron and those of the fcc structure (i.e., 360 deg.–5 × 70.5 deg.).

This behaviour is very similar to the observations made for multiply-twinned regions in the case of ISRO-mediated nucleation [8,16]. However, in this case, it happens during growth. It means that, rather than having the fcc phase nucleating from iQC or ISRO motifs, here we have rather an fcc phase growing on which ISRO motifs attach, then leading to the formation of a “new” fcc grain with a twinning relationship with the parent grain. As explained in a forthcoming paper [18], the formation of a twin during growth induced by ISRO motifs in the liquid will be called “ISRO-induced stacking fault”, and will be further discussed in Section 4.

**Figure 4:**
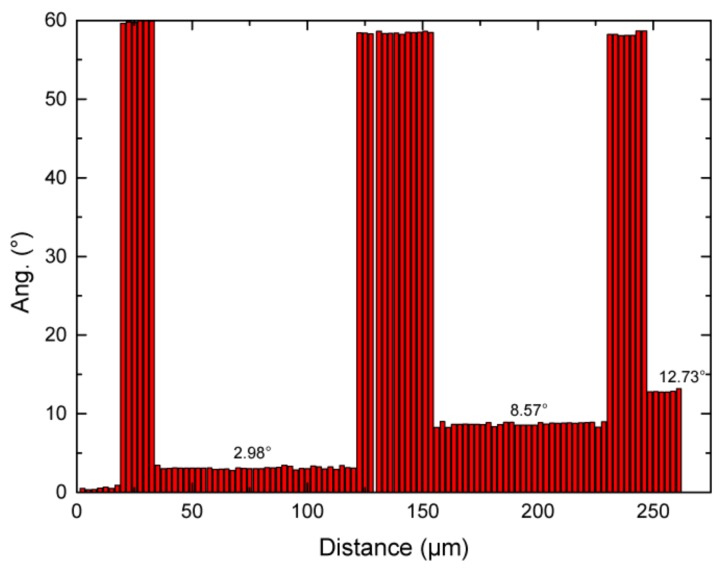
Relative misorientation angles along the red dashed-line of Figure 3, from left to right. The angle corresponds to the rotation between twinned or untwinned regions with respect to the initial region of the feathery grain.

These ISRO-induced stacking faults could also be responsible of the fan-shape and gradual misorientation of twinned dendrites within a feathery grain. In Figure 4, the misorientation along the red dashed line shown in Figure 3 has been measured. Taking the initial orientation measured on the left part of the line as a reference, the angle measured along the line corresponds to the rotation necessary to bring this part into coincidence with the reference, i.e., a perfect twin corresponds to an angle of 60 deg. (around the common ⟨111⟩ direction). As can be seen, besides the jumps between twinned and untwinned parts of the specimen, a small but systematic angular deviation is observed: from 0 deg., the first twinned part is at 60 deg., then the next untwinned region has a rotation angle of about 2.98 deg. with respect to the reference. After the next twinned region (which is not exactly at 60 deg. from the reference), the next untwinned part has a misorientation angle of 8.57 deg. with respect to the reference, i.e., a difference of 5.6 deg. with the previous untwinned region. Finally, after another twinned region, the final untwinned region has a rotation of 12.73 deg. with respect to the reference, or 4.2 deg. with respect to the previous one.

It is interesting to note that these relative misorientations (3, 5.6 and 4.2 deg.) are fairly close to multiples of the mismatch angles between the planes of the tetrahedra within an icosahedron and those of {111} planes: 1.47, 2.94, 4.41, 5.88 and 7.35 deg. In other words, a misorientation of 3 deg. corresponds to two mismatches, 5.6 deg. to four mismatches, and 4.2 deg. to three mismatches. The largest mismatch corresponding to five mismatches was measured between regions (1) and (2) of Figure 3, a value that was defined by Kurtuldu et al. as a near-twin relationship during iQC-mediated nucleation under nearly zero thermal gradient [8], and corresponding to the 7.35 deg aperture default angle associated with the decahedral configuration of 5 fcc tetrahedra.

## 4. Discussion

Based on the DS results of Al-Zn alloys presented in the previous section, it is clear that minor Cr additions influence the columnar microstructure in several ways. Although “regular” dendritic grains clearly exhibit a ⟨100⟩ texture in the FFD experiment with or without Cr, Kurtuldu et al. [3] have shown that, at lower speed, dendrite trunks grow along ⟨110⟩ with Cr, and ⟨100⟩ without Cr. This Cr-induced change of dendrite growth direction, from ⟨110⟩ at low speed to ⟨100⟩ at high speed, is clearly linked to an attachment kinetics effect rather than a modification of the solid–liquid interfacial energy. Kurtuldu et al. [3] have interpreted their result in terms of density of ISRO motifs in the liquid and time for them to attach to the growing fcc phase. Cr being a peritectic-type element, the density of Cr in the liquid ahead of a dendrite tip decreases, and so does the density of ISRO motifs. It is therefore not too surprising that ⟨100⟩ growth directions of “regular” grains are retrieved at high growth rate (typically > 1 mm/s) and our FFD results confirm that.

However, at the same time, under the rapid solidification conditions of the FFD experiment, we have shown that Cr-addition favors the formation of feathery grains. It has to be mentioned that, out of three experiments for each alloy, all of those containing Cr exhibit feathery grains while binary Al-Zn alloy have shown only regular columnar grains. This also confirms the results of [3] obtained for DS samples: they observed feathery grains in only one out of ten specimens without Cr, while with Cr eleven specimens out of twelve exhibited twinned dendrites. As illustrated in Figure 3, nucleation of a twinned dendrite seems to occur when a “regular” growing crystal has one direction ⟨110⟩ closely aligned with the direction of the thermal gradient. The growth of a twinned dendrite along this direction might then be kinetically favorable, i.e., lower undercooling, as compared to a “regular” dendrite growing along 〈100〉. This is especially the case of grain 1 in Figure 3, for which the closest 〈100〉 direction makes an angle of 25 deg. with respect to the thermal gradient. Its dendrite tip velocity, v^*^_⟨100⟩_, would be equal to *v_Liq_* / cos(25) = 1.1 *v_Liq_*, where *v_Liq_* is the velocity of the liquidus isotherm. This 10% increase in velocity further increases its undercooling compared to a well oriented regular ⟨100⟩ dendrite (or ⟨110⟩ twinned dendrite).

**Figure 5:**
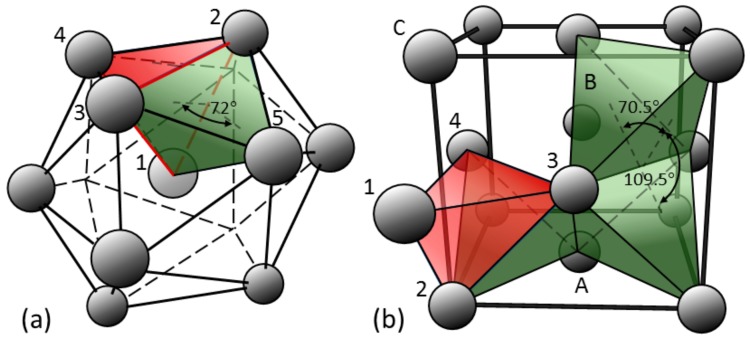
(**a**) arrangement of twenty slightly distorted tetrahedra in the icosahedron; (**b**) three {111} regular tetrahedra (in green) out of the eight within the unit fcc cell, and attachment of one of the tetrahedron of an ISRO motif (in red) creating a stacking fault.

While the growth of a twinned dendrite has a kinetic advantage, how does it nucleate? Figure 5 illustrates what can happen when ISRO occurs in the liquid. It has first to be recognized that {111} tetrahedra of the fcc structure touch each other via edges or vertices (Figure 5b). The angles between these planes are either 70.5 or 109. 5 deg. In Figure 5a, the twenty tetrahedra of an icosahedron look very much like {111} tetrahedra of the fcc structure, except for two key points: (i) since the central atom, Cr in the case of Al-Zn, is slightly smaller than the twelve surrounding atoms, the tetrahedra slightly distorted. The internal planes make an angle of 72 deg., while these planes with external triangular facet of the icosahedron make an angle of 69 deg.; (ii) these tetrahedra are linked via faces.

Consequently, when one icosahedral motif attaches to the growing fcc phase, it most probably does it via a facet: as shown in (b), the red tetrahedron (supposedly one element of the icosahedron) shares atoms 2-3-4 with the fcc phase and a stacking fault is induced on this plane. In a way similar to ISRO- or iQC-mediated nucleation, this mechanism could be called “ISRO-induced stacking fault”. When the growth rate is fast enough, this stacking fault has no time to anneal behind the solid–liquid interface and further fcc growth from this “ISRO nucleation center” leads to a twinned structure. This mechanism can explain both the nucleation and growth of a feathery grain, provided that local crystallography of the growing twinned dendrite is favorable to such ISRO-induced stacking fault with respect to the thermal gradient. Please note that, in the configuration shown in Figure 5 where Cr is atom 1, the angle between planes 2-3-4 and the other red planes of the tetrahedron is only 69 deg., compared to 70.5 deg. for the green {111} planes of the regular fcc structure. This small gap of 1.5 deg. is responsible for the near-twin relationship, i.e., a perfect twin relationship plus a rotation of 1.5 deg. around a common ⟨110⟩ direction.

## 5. Conclusions

Using high purity materials, it has been shown that minor Cr additions leads to the formation of feathery grain in Al-Zn alloys rapidly solidified using the Free-Falling Droplet experiment. This confirms the observations of Kurtuldu et al. [3] on DS experiments. EBSD analysis performed in this work has allowed to show that nucleation and propagation of twinned dendrites leading to feathery grains in Al-Zn alloys with 1000 ppm Cr added can be associated with near-twin orientation relationships, i.e., a {111} twin orientation relationship plus a rotation of a few deg. around a common ⟨110⟩ direction. In a way similar to iQC- or ISRO-mediated nucleation where the fcc phase forms on an icosahedron template, the present observations support the mechanism of an ISRO-induced stacking fault. When an icosahedral motif attaches to the growing fcc phase, this induces a stacking fault but with a 1.5 deg. misorientation since the planes of the tetrahedra belonging to the icosahedral motif make either an angle of 72 deg. (internal planes) or 69 deg. (angle between an internal plane and the corresponding facet of the icosahedron), while the {111} planes of the fcc structure make an angle of 70.5 deg. Depending on how the ISRO motif attaches on the {111} planes of the growing fcc phase, the rotation along ⟨110⟩ can be +1.5 or −1.5 deg. Further growth of the fcc phase from this ISRO motif leads to the formation of a twinned dendrite.
